# Physiological Regularity and Synchrony in Individuals with Gaming Disorder

**DOI:** 10.3390/e26090769

**Published:** 2024-09-08

**Authors:** Hung-Ming Chi, Tzu-Chien Hsiao

**Affiliations:** 1 Department of Medical Informatics, College of Health Care and Management, Chung Shan Medical University, Taichung 40201, Taiwan; 2 Department of Computer Science, College of Computer Science, National Yang Ming Chiao Tung University, Hsinchu 30010, Taiwan; labview@nycu.edu.tw; 3 Institute of Biomedical Engineering, College of Electrical and Computer Engineering, National Yang Ming Chiao Tung University, Hsinchu 30010, Taiwan

**Keywords:** abdominal wall movement, gaming disorder, physiological complexity, pulse wave, sample entropy, thoracic wall movement, vascular-respiratory coupling

## Abstract

Individuals with gaming disorder (GD) show emotional dysregulation and autonomic dysfunction in daily life. Although studies have shown that the relaxation method of breathing exercise (BE) improves cardiopulmonary synchrony, the physiological regularity and synchrony of GD remain unclear. In this study, we investigated the regularities of pulse wave (PW), thoracic wall movement (TWM), and abdominal wall movement (AWM) using sample entropy (SE) and assessed the vascular-respiratory and TWM-AWM synchrony using cross-sample entropy (CSE). Twenty individuals with GD and 26 healthy control (HC) individuals participated in baseline, gaming, and recovery stages, both before and after BEs. The results showed that both groups had significantly higher SE_TWM_, SE_AWM_, and CSE_TWM-AWM_ during gaming than baseline. Before BE, CSE_PW-TWM_ and CSE_PW-AWM_ during gaming were considerably higher in the GD group than in the HC group. Compared to before BE, both groups had decreased SE_TWM_ and CSE_TWM-AWM_ during gaming, particularly in the HC group. Online gaming may induce pulse wave and respiratory irregularities, as well as thoracic–abdominal wall movement asynchrony. Individuals with GD who engage in prolonged gaming periods may exhibit lower vascular–respiratory synchrony compared to the HC group. SE_TWM_, SE_AWM_, CSE_TWM-AWM_, CSE_PW-TWM_, and CSE_PW-AWM_ may serve as biomarkers for assessing the risk of GD. BE may improve TWM regularity and vascular–respiratory synchrony during gaming, potentially alleviating addictive behavior.

## 1. Introduction

The inability to properly control the usage of online gaming can cause adverse psychophysiological consequences, such as emotional dysregulation, decision-making biases, attention deficits, loss of control, and impulsive behaviors [1]. Due to these consequences, gamers give up other leisure activities, become estranged from family and friends, experiencing lower academic performance and declining work productivity. American psychologists identified these conditions as indicative of internet gaming disorder [2], while the World Health Organization announced it as gaming disorder (GD) under the category of impulse control disorders [3]. The global focus on GD is gradually increasing. The National Health Service in the United Kingdom has established the National Centre for Gaming Disorders to assess and treat GD cases [4]. Additionally, the government of Kagawa Prefecture in Japan has enacted an ordinance restricting online game usage time for minors [5]. Individuals with GD are immersed in gaming environments for prolonged periods, and their physiological regulatory patterns may serve as objective indices (including both linear and nonlinear aspects) to assist in assessing and preventing GD.

Analysis of heart rate variability (HRV) in both the time and frequency domains is commonly used to measure the degree of variation in the intervals between heartbeats in individuals with GD. During rest, the GD group showed higher heart rates than regular gamers [6], but lower root mean square of successive differences and high-frequency HRV [7,8]. The GD groups also exhibited significantly lower high-frequency HRV during gaming than baseline [9,10]. Due to reduced cognitive control, online gaming resulted in a higher low-frequency to high-frequency ratio [11] and lower high-frequency HRV [12] in those with GD compared to regular gamers. These results suggested autonomic dysfunction in individuals with GD, characterized by either parasympathetic withdrawal or sympathetic dominance. However, these HRV responses are linear indices and are insufficient for a comprehensive exploration of psychophysiological regulation.

Entropy, a nonlinear index, indicates physiological irregularity or complexity in patients with mental disorders. Compared to healthy control (HC) individuals, patients with schizophrenia exhibited irregular coordination between the cardiovascular and respiratory systems [13,14]. Individuals with depressive symptoms showed higher heart rate complexity and lower cardiorespiratory coupling, suggesting central autonomic dysregulation [15]. Cardiovascular and respiratory complexities were also observed during stress, with mental stress reducing cardiovascular–respiratory coupling [16] and increasing peripheral blood volume changes, blood pressure, and respiratory irregularities [17], indicating vagus nerve dysregulation. After treatment or breathing exercise (BE), patients with mental disorders showed increased peripheral blood volume change regularity [18] and enhanced cardiorespiratory synchrony [19]. Since entropy has been used to estimate physiological regularity, studying the entropy of cardiovascular and respiratory responses in individuals with GD is valuable.

Breathing can both manifest and influence autonomic responses. Negative emotional stimuli have been shown to drive thoracic wall movement (TWM) [20]. After a BE, abdominal wall movement (AWM) became more complex than TWM [21,22]. Peripheral vasoconstriction may stabilize blood pressure under respiratory regulation [23]. To explore physiological regularity and synchrony in individuals with GD, this study observed the pulse wave, TWM, and AWM regularities and vascular–respiratory coupling during online gaming before and after BEs. We also compared the physiological responses of the GD and HC groups. This study proposes three hypotheses. First, physiological regularity and synchrony would differ between the baseline and gaming stages. Second, the GD group would show different physiological regularity and synchrony compared to the HC group. Third, physiological regularity and synchrony would vary before and after BEs. Long-term maintenance of addictive behaviors may lead to pulse waves and respiratory irregularities as well as vascular–respiratory decoupling in individuals with GD. These physiological responses may serve as biomarkers to assess and prevent GD. Moreover, BE may assist cognitive behavioral therapy in improving vascular–respiratory coupling and alleviating addictive behavior.

## 2. Materials and Methods

### 2.1. Participant

Since most gamers are between the ages of 20 and 39 [24,25], online games are more likely to affect the psychophysiological regulation of university students. This study recruited participants aged 20–40 from universities in Hsinchu, Taiwan. The inclusion criteria were as follows: (1) engagement in multiplayer role-playing games, (2) absence of cardiovascular diseases and depressive symptoms, and (3) score above 63 on the Chen Internet Addiction Scale (CIAS) [26] and above four on the Internet Gaming Disorder Questionnaire (IGDQ) [27] for the GD group. Participants who did not meet these CIAS and IGDQ scores were assigned to the HC group. A total of 117 university students were screened, and 46 were included in this study, comprising 20 in the GD group (17 males and three females; mean age 22.90 ± 3.19 years) and 26 in the HC group (23 males and three females; mean age 23.38 ± 2.59 years). Since the participants were recruited from universities primarily focused on science-related programs, the majority were male.

The Research Ethics Committee for Human Subject Protection of National Chiao Tung University approved the experimental design and participant recruitment. All procedures used in this study were based on good clinical practice guidelines and were conducted under the latest version of the Helsinki Declaration. After we explained the experimental methods to each participant, they signed an informed consent form.

### 2.2. Questionnaire

The CIAS is a 26-item questionnaire designed to assess compulsive, tolerance, and withdrawal symptoms related to internet addiction, as well as negative consequences on time management and health due to addiction [26]. Each item is rated on a scale from 1 (strong disagreement) to 4 (strong agreement), with higher scores indicating more severe addiction symptoms. A cutoff point of 63/64 was set up, meaning that scores of 64 or higher were classified as indicative of internet addiction [28]. The total score on the CIAS ranges from 26 to 104, with a reliability coefficient of 0.87 for the 46 participants in this study. The IGDQ consists of 9 items corresponding to the diagnostic criteria of Internet Gaming Disorder in the Diagnostic and Statistical Manual of Mental Disorders, fifth edition [27]. The IGDQ assesses the risk of GD by asking participants about their online gaming experiences, with responses recorded as yes or no. A cutoff point of 4/5 was used, meaning that scores of 5 or higher were classified as indicative of GD [2]. The IGD scores range from 0 to 9. In this study, Cronbach’s alpha was measured as 0.91. The Self-Assessment Manikin (SAM), a scale comprising two items, was utilized to evaluate the emotional valence and arousal induced by cue stimuli [29]. The rating scale for each item ranges from 1 to 9. For emotional valence, a score of 5 represents a neutral emotion; scores below 5 indicate negative emotions; and scores above 5 indicate positive emotions. For emotional arousal, higher scores reflect greater emotional intensity.

### 2.3. Physiological Signal

Respiratory signals were measured using respiratory inductance plethysmography (RIPmate Inductance Belt, Adult, Ambu, Ballerup, Denmark), with belts placed around the thorax and abdomen to record TWM and AWM, respectively. Pulse wave signals were obtained using a noninvasive blood pressure monitoring device (NIBP-100D, Biopac Inc., Goleta, CA, USA). This device utilized an inflatable adult-sized blood pressure cuff (23–33 cm) positioned on the left upper arm, and a medium (18–24 mm) or large (24–28 mm) double-cuff finger sensors placed on the left middle and index fingers to measure pulse wave changes. To measure respiratory muscle contractions [30,31,32] and simultaneously collect pulse wave signals, the TWM, ATM, and pulse wave signals were recorded using a DAQCard (USB 6255, 16-bit resolution, NI, Austin, TX, USA) at a sampling rate of 1000 Hz and a range of ± 5 volts. Then, these signals were transferred to a personal computer (Acer M2610, Intel Core i3-2120, 4 GB DDR3-1066, Windows 10 Professional 64-bit, Taipei, Taiwan).

### 2.4. Experimental Procedure

**Figure 1:**
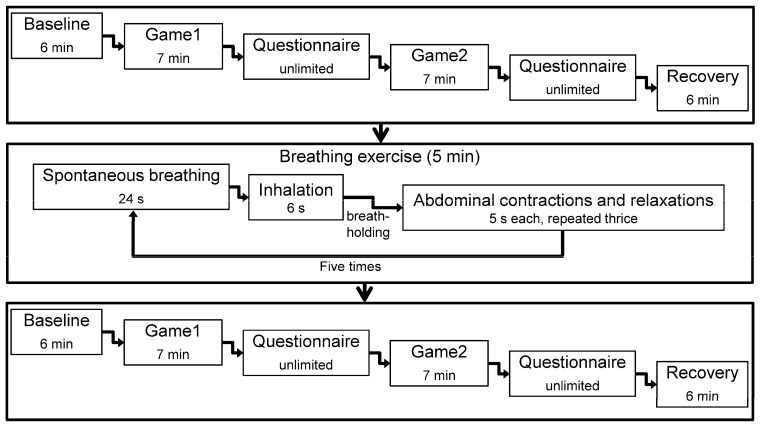
The experimental procedure.

Participants were asked to avoid vigorous exercise and to refrain from consuming caffeine-containing beverages and food before the experiment. The environmental temperature was maintained between 23 and 27 degrees Celsius. During the experiment, each participant sat on a chair with back support, faced a computer screen, and minimized movement. The participant sequentially completed the baseline, game1, game2, and recovery stages (Figure 1). In the baseline stage, the participant relaxed his body and calmed his mind by watching a picture of a gray background with a black dot at the center for 6 min. In the game1 and game2 stages, the participant played KartRider (Nexon Co., Ltd., Seoul, Republic of Korea) at levels two and four, respectively. Level four was more challenging than level two. Each game stage lasted 7 min, during which the participant competed against seven robots. After each game stage, the participant filled out the SAM questionnaire. In the recovery stage, the participant again watched the picture of a gray background with a black dot at the center for 6 min to relax his psychophysiological responses. Subsequently, the participant performed an iso-volume maneuver training as BE [22,33]. The BE procedure was as follows: first, the participant completed 24 s of spontaneous breathing. Second, he inhaled for 6 s before holding his breath. Third, he maintained lung capacity to focus on forceful abdominal contractions and relaxations for 5 s each, repeated thrice. The entire BE process was repeated five times, totaling 5 min. The participant’s breathing was monitored to confirm the correct execution of BE. Then, the participant completed the baseline, game1, game2, and recovery stages again. TWM, AWM, and pulse wave signals were measured throughout the experiment.

### 2.5. Signal Processing

**Figure 2:**
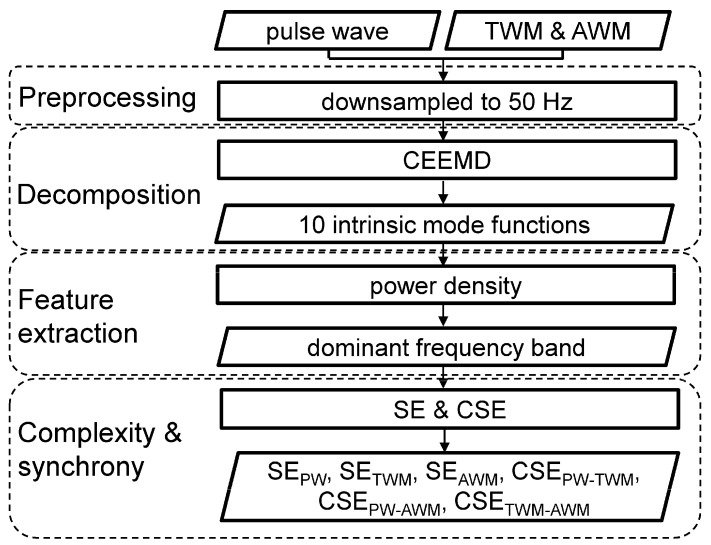
The framework of signal processing. AWM, abdominal wall movement; CEEMD, complementary ensemble empirical mode decomposition; TWM, thoracic wall movement; CSE, cross-sample entropy; SE sample entropy; CSE_PW-TWM_, CSE of between pulse wave and TWM; CSE_PW-AWM_, CSE of between pulse wave and AWM; CSE_TWM-AWM_, CSE of between TWM and AWM; SE_AWM_, SE of AWM; SE_PW_, SE of pulse wave; SE_TWM_, SE of TWM.

Processing physiological signals involves preprocessing, decomposition, feature extraction, and complexity and synchrony calculations stages (Figure 2).

In the preprocessing stage, previous studies showed that downsampling acquired signals from 1000 Hz to 50 Hz can reduce processing time [30,31,32,33]. In the decomposition stage, complementary ensemble empirical mode decomposition (CEEMD) [34] as an adaptive filter was utilized to decompose the nonlinear and non-stationary signals into intrinsic mode functions (IMFs), representing oscillatory patterns from high to low frequencies. The relationship between the signal and its IMFs is given by:

(1) $$x\left(t\right)=\sum_{i=1}^{10}{IMF}_{i}(t)+r\left(t\right),$$

where $x\left(t\right)$ denotes the signal at 50 Hz, ${IMF}_{i}(t)$ is the *i*th IMF, and $r\left(t\right)$ is the final residue after obtaining the 10 IMFs.

In the feature extraction stage, the energy density method was used to calculate the energy of each IMF [26], expressed as follows:

(2) $$P_{i}=\frac{1}{N}\sum_{t=1}^{N}{IMF}_{i}^{2}\left(t\right),$$

where $P_{i}$ and *N* denote the energy of *i*th IMF and the signal length, respectively. The IMF with the highest energy was identified as the dominant component (DC) of $x\left(t\right)$.

In the complexity and synchrony calculations stage, sample entropy (SE) was utilized to estimate the irregularity and complexity of a single time series [35], defined as follows:

(3) $$SE\left(m,\;r,\;N\right)=-ln\left(\frac{A^{m}(r)}{B^{m}(r)}\right),$$

where *m* represents the dimension of two time series undergoing comparison, and r represents the tolerance level for accepting matches. $B^{m}(r)$ and $A^{m}(r)$ denote the conditional probabilities of two time series matching for *m* and *m* + 1 points, respectively. The higher SE value indicates more irregularity and complexity in the signal. cross-sample entropy (CSE) was utilized to assess the synchrony or similarity between two time series υ and μ [35], described as follows:

(4) $$CSE\left(m,\;r,\;N\right)=-ln\left\{\frac{A^{m}(r)\left(\upsilon \left\|\mu \right.\right)}{B^{m}(r)\left(\upsilon \left\|\mu \right.\right)}\right\},$$

where $B^{m}(r)\left(\upsilon \left\|\mu \right.\right)$ and $A^{m}(r)\left(\upsilon \left\|\mu \right.\right)$ denote the conditional probabilities of υ and μ templates matching for *m* and *m* + 1 points, respectively. The higher CSE value indicates asynchrony or decoupling between the two physiological signals. In this study, the DC was normalized by subtracting its mean and dividing by its standard deviation to reduce individual differences. Subsequently, the SE method was applied to evaluate the regularities of the normalized DCs of the pulse wave, TWM, and AWM signals, denoted as SE_PW_, SE_TWM_, and SE_AWM_, respectively. Additionally, CSE was used to calculate the coupling between the normalized DCs of the pulse wave and TWM signals, the normalized DCs of the pulse wave and AWM signals, and the normalized DCs of TWM and AWM signals, denoted as CSE_PW-TWM_, CSE_PW-AWM_, and CSE_TWM-AWM_, respectively. The parameters *m* and *r* for both SE and CSE methods were set to 2 and 0.15 [36], respectively. All signal processing was performed using the LabVIEW program (2019 version, NI, Austin, TX, USA).

### 2.6. Statistical Analysis

Based on previous GD studies involving 41 to 68 participants [8,9,10,11,12], our sample size of 46 was sufficient to observe physiological differences between the GD and HC groups. Chi-square tests were conducted to determine the association between genders in the GD and HC groups. The Wilcoxon signed-rank test was used to estimate differences in SAM scores between game1 and game2, as well as before and after BEs. The Mann–Whitney *U* test was utilized to evaluate differences in questionnaire scores and the physiological complexity and synchrony between the GD and HC groups.

To investigate differences between the two groups during the various stages before and after BEs, a factorial analysis of variance (ANOVA) was performed with groups (GD and HC) as a between-subjects factor, and stages (baseline, game1, game2, and recovery) and BE (before and after BEs) as within-subjects factors. Bonferroni correction was implemented to address multiple comparison problems. Additionally, the Wilcoxon signed-rank test was applied to examine differences in the physiological complexity and synchrony between the baseline and the other three stages, as well as before and after BEs. All statistical tests were two-tailed; *p* < 0.05 was considered statistically significant. Statistical analysis was conducted using SPSS version 21.0 (SPSS, Chicago, IL, USA).

## 3. Results

### 3.1. Questionnaire

No significant differences in gender (χ^2^ = 0.12, *p* = 0.730) and age (*Z* = −1.58, *p* = 0.114) were observed between the GD and HC groups. Significant differences were found between the CIAS (CIAS_GD_ = 71.95 ± 6.02, CIAS_HC_ = 51.00 ± 8.28, *Z* = −5.77, *p* < 0.001) and IGDQ scores (IGDQ_GD_ = 5.55 ± 1.00, IGDQ_HC_ = 1.15 ± 1.05, *Z* = −5.89, *p* < 0.001) of the two groups.

Table 1 presents the SAM scores of participants after playing online games. Before BE, the emotional valence of the GD group in game1 was significantly higher than in game2 (*Z* = −2.250, *p* = 0.024), indicating that game1 elicited more positive emotions. After BE, the emotional valence of the HC group at game1 was significantly higher than in game2 (*Z* = −2.123, *p* = 0.034). Moreover, the emotional valence of the GD group in game1 after BE was significantly lower than that of the HC group (*Z* = −2.385, *p* = 0.017). The emotional valence (*Z* = −2.430, *p* = 0.015) and arousal (*Z* = −2.072, *p* = 0.038) of the HC group in game1 were significantly higher after BE compared to before BE.

### 3.2. Factorial ANOVA

**Figure 3:**
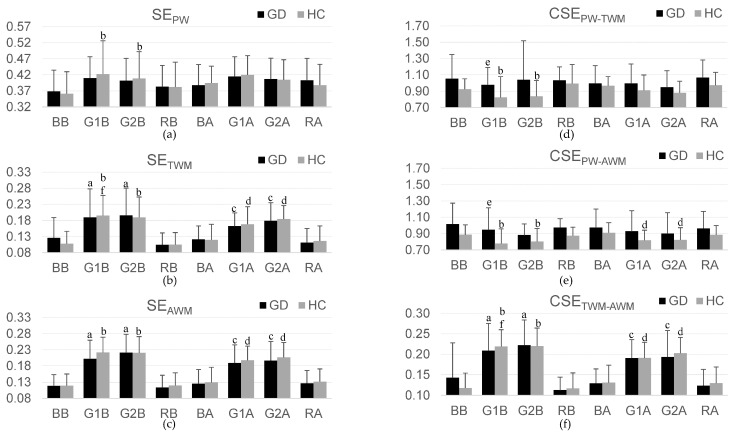
Bar graph showing the mean and standard deviation of physiological complexity and synchrony for the gaming disorder (GD) and healthy control (HC) groups: (**a**) sample entropy (SE) of pulse wave signal (SE_PW_); (**b**) SE of thoracic wall movement signal (SE_TWM_); (**c**) SE of abdominal wall movement signal (SE_AWM_); (**d**) cross-sample entropy (CSE) of between pulse wave and thoracic wall movement signals (CSE_PW-TWM_); (**e**) CSE of between pulse wave and abdominal wall movement signals (CSE_PW-AWM_); (**f**) CSE of between thoracic and abdominal wall movement signals (CSE_TWM-AWM_) values. BB, baseline before breathing exercise (BE); G1B, game1 before BE; G2B, game2 before BE; RB, recovery before BE; BA, baseline after BE; G1A, game1 after BE; G2B, game2 after BE; RA, recovery after BE. ^a^ *p* < 0.05 compared BB with the other three stages before BE for the GD group; ^b^ *p* < 0.05 compared BB with the other three stages before BE for the HC group; ^c^ *p* < 0.05 compared BA with the other three stages after BE for the GD group; ^d^ *p* < 0.05 compared BA with the other three stages after BE for HC group; ^e^ *p* < 0.05 compared GD with HC groups; ^f^ *p* < 0.05 compared before BE with after BE.

Figure 3 displays bar graphs of the mean and standard deviation of SE_PW_, SE_TWM_, SE_AWM_, CSE_PW-TWM_, CSE_PW-AWM_, and CSE_TWM-AWM_ for the GD and HC groups during baseline, game1, game2, and recovery stages before and after BEs. The numerical values (mean and standard deviation) of Figure 3 are included in Table S1.

Table 2 presents the statistical results of the factorial ANOVA. The results demonstrated significant differences in all physiological complexities and couplings across different experimental stages, except for CSE_PW-TWM_. CSE_PW-TWM_ and CSE_PW-AWM_ exhibited significant differences between the HC and GD groups. No significant physiological differences were observed between before and after BEs. Only SE_TWM_ demonstrated a significant interaction effect between experimental stages and BE.

### 3.3. The Differences among Experimental Stages 

Before BE, the GD group showed significantly lower SE_TWM_, SE_AWM_, and CSE_TWM-AWM_ during the baseline than during the game stages (game1 stage: SE_TWM_ (*Z* = −2.616, *p* = 0.009), SE_AWM_ (*Z* = −2.777, *p* = 0.005), and CSE_TWM-AWM_ (*Z* = −3.058, *p* = 0.002); game2 stage: SE_TWM_ (*Z* = −3.501, *p* < 0.001), SE_AWM_ (*Z* = −3.783, *p* < 0.001), and CSE_TWM-AWM_ (*Z* = −3.058, *p* = 0.002)). The HC group also exhibited significantly lower SE_PW_, SE_TWM_, SE_AWM_, and CSE_TWM-AWM_ during the baseline than during the game stages (game1 stage: SE_PW_ (*Z* = −2.451, *p* = 0.014), SE_TWM_ (*Z* = −4.076, *p* < 0.001), SE_AWM_ (*Z* = −4.407, *p* < 0.001), and CSE_TWM-AWM_ (*Z* = −4.197, *p* < 0.001); game2 stage: SE_PW_ (*Z* = −2.654, *p* = 0.008), SE_TWM_ (*Z* = −4.045, *p* < 0.001), SE_AWM_ (*Z* = −4.457, *p* < 0.001), and CSE_TWM-AWM_ (*Z* = −4.197, *p* < 0.001)), but higher CSE_PW-TWM_ and CSE_PW-AWM_ (game1 stage: CSE_PW-TWM_ (*Z* = −2.677, *p* = 0.007) and CSE_PW-AWM_ (*Z* = −2.129, *p* = 0.033); game2 stage: CSE_PW-TWM_ (*Z* = −3.187, *p* = 0.001) and CSE_PW-AWM_ (*Z* = −2.451, *p* = 0.014)). Moreover, the GD group showed significantly higher CSE_PW-TWM_ (Z = −2.594, *p* = 0.009) and CSE_PW-AWM_ (Z = −2.413, *p* = 0.016) during game1 compared to the HC group.

After BE, the GD group showed significantly lower SE_TWM_, SE_AWM_, and CSE_TWM-AWM_ during the baseline than during the game stages (game1 stage: SE_TWM_ (*Z* = −3.441, *p* = 0.001), SE_AWM_ (*Z* = −4.254, *p* < 0.001), and CSE_TWM-AWM_ (*Z* = −4.178, *p* < 0.001); game2: SE_TWM_ (*Z* = −3.501, *p* < 0.001), SE_AWM_ (*Z* = −3.743, *p* < 0.001), and CSE_TWM-AWM_ (*Z* = −3.380, *p* < 0.001)). The HC group also exhibited significantly lower SE_TWM_, SE_AWM_, and CSE_TWM-AWM_ during the baseline than during the game stages (game1 stage: SE_TWM_ (*Z* = −2.857, *p* = 0.004), SE_AWM_ (*Z* = −3.662, *p* < 0.001), and CSE_TWM-AWM_ (*Z* = −3.783, *p* < 0.001); game2: SE_TWM_ (*Z* = −4.127, *p* < 0.001), SE_AWM_ (*Z* = −4.457, *p* < 0.001), and CSE_TWM-AWM_ (*Z* = −4.432, *p* < 0.001)), but higher CSE_PW-AWM_ (stimuli1: *Z* = −3.264, *p* = 0.001 and stimuli2: *Z* = −2.857, *p* = 0.004).

### 3.4. The Regularity and Synchrony Difference after BE

The GD group did not show significant differences in physiological regularity and synchrony after BE. However, the HC group exhibited significantly lower SE_TWM_ (*Z* = −3.081, *p* = 0.002) and CSE_TWM-AWM_ (*Z* = −3.404, *p* = 0.001) during game1 stage after BE.

## 4. Discussion

This is the first study to investigate pulse wave, TWM, and AWM regularities, as well as vascular–respiratory coupling during online gaming in university students with GD using nonlinear methods and indices. Furthermore, we observed the effect of BE on these regularities and couplings to explore the effectiveness of BE in reducing autonomic dysfunction.

We hypothesized that the physiological regularity and synchrony would show differences between baseline and gaming stages. We found that before and after BEs, SE_TWM_, SE_AWM_, and CSE_TWM-AWM_ for both GD and HC groups during game1 and game2 stages were significantly higher than during baseline, while CSE_PW-AWM_ of the HC group was significantly lower. These findings partially support our hypothesis and agree with previous studies showing that facing mathematical stress or playing games increased respiratory and cardiovascular irregularities compared to rest [16,37]. Negative picture stimuli increased the TWM-to-AWM ratio [20]. The possible explanation for these findings may be that online games, with complex auditory and visual stimuli, activate the central and autonomic nervous systems [38]. The central nervous system drives diverse emotional responses and cognitive controls [39]. Young adults with GD were mainly focused on pursuing game rewards [40], and winning a game triggered positive emotions, whereas failure induced stress and negative emotions. On the other hand, the autonomic nervous system comprises the sympathetic and parasympathetic nervous systems. Online games may activate the sympathetic or inhibit the parasympathetic nervous system, causing increased physiological responses such as heart rate, respiratory rate, or blood pressure, especially in young adults with GD [41]. Additionally, cardiorespiratory interactions may suppress blood flow variability and improve cardiac efficiency [23]. Compared to baseline, cardiorespiratory synchrony increased when gamers watched game videos [42]. Gamers may increase vascular–respiratory coupling to maintain homeostasis during gaming. Therefore, we infer that online games elicit dynamic changes in emotional valence and intensity to increase vascular and respiratory complexities, thoracic–abdominal wall movement asynchrony, and vascular–respiratory coupling, especially among university gamers. These physiological complexities and synchronies can be quantified using SE and CSE methods. More data are needed to validate this inference.

This study also hypothesized that individuals with GD would show different physiological complexity and synchrony to the HC group. The results exhibited that university students with GD exhibited a significantly lower emotional valence during the game1 stage after BE compared to the HC group. Additionally, CSE_PW-TWM_ and CSE_PW-AWM_ of university students with GD were significantly higher than those of the HC group, particularly before BE. These findings partially support our hypothesis. Consistent with the literature, Chang et al. proposed that university students with GD showed higher cardiopulmonary asynchrony compared to the HC group [43]. This asynchrony may be attributed to autonomic dysfunction, where young students with GD increased sympathetic activity or inhibited parasympathetic activity [8,44], leading to irregularities in cardiopulmonary coordination [43]. Individuals with GD often experienced emotional dysregulation and had a high risk of depressive and anxiety symptoms [45,46]. Patients with mental disorders may have cardiovascular damage and vagus nerve dysfunction, causing increased complexity in the interaction between heart and respiration [13,14,15,46]. Therefore, we infer that university students with GD show emotional dysregulation and autonomic dysfunction, leading to decreased vascular–TWM and vascular–AWM synchrony. Entropy, as a nonlinear index, can assist questionnaires in assessing the risk of GD.

We hypothesized that the physiological regularity would vary between before and after BEs. The HC and GD groups showed decreases in SE_TWM_ and CSE_TWM-AWM_ during the game1 stage after BE, with the HC group exhibiting statistically significant changes. Neither group showed a significant reduction in CSE_PW-TWM_ or CSE_PW-AWM_ after BE. These findings partially support our hypothesis. However, in contrast to the previous studies, long-term BE in patients with mental disorders (e.g., over 15 days) significantly increases cardiorespiratory synchrony [19] and decreases heart rate [47]. BE, especially at six cycles per minute, influences the cerebral cortex to relax the body and enhance vagus nerve activity [48]. The vagus nerve activation increases parasympathetic activity, raising heart rate variability, brain–heart interaction, and cardiorespiratory coupling [49]. The inconsistencies in our findings may be due to individual variability in responses to short-term BE, suggesting that long-term BE may be necessary [50,51]. The small sample size may also affect the statistically significant differences, particularly in the GD group. Although the short-term BE did not significantly improve cardiorespiratory coupling, young students with GD significantly increased total power and low-frequency HRV after 10 min of BE [52]. Research suggests that BE can assist cognitive–behavioral therapy [53] or meditation [54] in alleviating game cravings and managing impulse control. Therefore, we infer that the duration of BE intervention and sample size are critical factors in observing the effect of BE on thoracic–abdominal wall movement and vascular–respiratory synchrony in individuals with GD.

Although the experiment data were acquired and analyzed under strict IRB approval, this study has several limitations. First, due to the limited sample size, we could not evaluate additional differences between the GD and HC groups, such as gender differences or variations among subgroups receiving clinical treatments. Second, psychiatrists did not conduct interviews with the participants. The risk of GD was assessed only through the CIAS and IGDQ, and these subjective results may yield screening bias. Third, a sampling rate of 1000 Hz was too high and may cause oversampling effects. A lower sampling rate, such as 200 Hz, should be used instead. Fourth, most studies divided patients with mental disorders into groups with clinical treatments and with BE [19] or into groups with and without BEs [47]. For this matter, we should recruit more participants and categorize those with GD into groups without and with long-term BEs in further study. Fifth, different online games may elicit varied physiological responses. We should investigate the effect of other popular games. Sixth, most participants were unfamiliar with the KartRider game, so they may have been unable to observe the real psychophysiological responses from the GD groups. The chosen game should be similar to those gamers habitually play. Finally, we did not investigate whether different downsampling rates affected the calculation of SE and CSE. Govindan et al. addressed downsampling issues by modifying the SE algorithm to include time delays between successive signals, improving physiological complexity evaluation [55]. We will use different SE algorithms to validate our study’s results in future work.

## 5. Conclusions

This study investigated pulse wave, TWM, and AWM regularities and vascular–respiratory coupling in university students with GD during online gaming, before and after BEs. We found that SE_TWM_, SE_AWM_, and CSE_TWM-AWM_ in the GD and HC groups during gaming before and after BEs were significantly higher than baseline, but CSE_PW-AWM_ in these with HC was lower. Before BE, the GD group showed significantly higher CSE_PW-TWM_ and CSE_PW-AWM_ during game1 stage than the HC group. Compared to before BE, both HC and GD groups decreased SE_TWM_ and CSE_TWM-AWM_ during game1 stage, with a significant decrease in the HC group. These findings suggest that online gaming increases TWM and AWM irregularities and thoracic–abdominal wall movement asynchrony, while enhancing vascular–respiratory coupling. Due to autonomic dysfunction, university students with GD exhibited lower vascular–respiratory coupling during online gaming compared to the HC group. SE_TWM_, SE_AWM_, CSE_TWM-AWM_, CSE_PW-TWM_, and CSE_PW-AWM_ may serve as objective indices for assessing the risk of GD. BE may be used to improve TWM regularity and vascular–respiratory synchrony during gaming, thereby enhancing autonomous function and reducing addiction symptoms. However, this positive effect of BE was observed only in the HC group, suggesting that similar benefits might be achievable in the GD group with longer BE training or larger sample sizes. Future research will explore various types of games and different SE algorithms to provide a more comprehensive understanding of the physiological regulation of individuals with GD.

## Figures and Tables

**Table 1:** The SAM scores (mean ± standard deviation).

Emotion		HC	GD
Before BE			
game1	Valence	6.04 ± 1.80 ^d^	6.05 ± 1.73 ^a^
	Arousal	6.04 ± 1.43 ^e^	6.70 ± 1.26
game2	Valence	5.77 ± 1.99	4.55 ± 2.16 ^a^
	Arousal	6.46 ± 1.58	7.05 ± 1.96
After BE			
game1	Valence	6.69 ± 1.72 ^b,c,d^	5.70 ± 1.63 ^c^
	Arousal	6.62 ± 1.47 ^e^	6.55 ± 1.99
game2	Valence	5.92 ± 1.87 ^b^	5.15 ± 1.93
	Arousal	6.65 ± 1.44	6.40 ± 1.93

BE, breathing exercise; GD, gaming disorder; HC, healthy control; ^a^ *p* < 0.05 significant difference in valence between game1 and game2 for the GD group; ^b^ *p* < 0.05 significant difference in valence between game1 and game2 for the HC group; ^c^ *p* < 0.05 significant difference in valence between the GD and HC groups; ^d^ *p* < 0.05 significant difference in valence between before and after BEs for the HC group; ^e^ *p* < 0.05 significant difference in arousal between before and after BEs for the HC group.

**Table 2:** Factorial ANOVA of physiological complexity and synchrony.

Parameter	Stage			BE			Group			Stage × BE			Stage × Group			BE × Group		
	$\eta_{p}^{2}$	*F*	*p*	$\eta_{p}^{2}$	*F*	*p*	$\eta_{p}^{2}$	*F*	*p*	$\eta_{p}^{2}$	*F*	*p*	$\eta_{p}^{2}$	*F*	*p*	$\eta_{p}^{2}$	*F*	*p*
SE_PW_	0.080	5.46	0.001 ^a^	0.009	1.88	0.172	<0.001	0.01	0.921	0.010	0.69	0.561	0.003	0.23	0.878	<0.001	0.06	0.811
SE_TWM_	0.311	33.43	<0.001 ^a^	0.003	1.08	0.299	<0.001	0.01	0.942	0.049	5.23	0.002 ^c^	0.014	1.46	0.224	0.001	0.48	0.491
SE_AWM_	0.618	84.78	<0.001 ^a^	0.002	0.63	0.427	0.004	1.57	0.211	0.020	2.69	0.046	0.002	0.24	0.867	<0.001	<0.01	0.978
CSE_PW-TWM_	0.521	3.48	0.016	0.004	0.07	0.789	0.860	17.27	<0.001 ^b^	0.070	0.47	0.704	0.070	0.47	0.702	0.087	1.74	0.188
CSE_PW-AWM_	0.535	5.68	0.001 ^a^	0.002	0.07	0.787	0.903	28.78	<0.001 ^b^	0.011	0.12	0.947	0.048	0.50	0.679	0.031	1.00	0.319
CSE_TWM-AWM_	0.569	83.21	<0.001 ^a^	0.006	2.84	0.093	<0.001	0.01	0.929	0.020	2.94	0.033	0.005	0.66	0.578	0.001	0.52	0.472

BE, breathing exercise; CSE_PW-TWM_, cross-sample entropy (CSE) of between pulse wave (PW) and thoracic wall movement (TWM) signals; CSE_PW-AWM_, CSE of between PW and abdominal wall movement (AWM) signals; CSE_TWM-AWM_, CSE of between TWM and AWM signals; SE_AWM_, sample entropy (SE) of AWM signal; SE_PW_, SE of PW signal; SE_TWM_, SE of TWM signal; ^a^ *p* < 0.008 after Bonferroni correction (0.05/6); ^b^ *p* < 0.05 after Bonferroni correction; ^c^ *p* < 0.006 after Bonferroni correction (0.05/8).

## Data Availability

The data presented in this study are available on request from the corresponding author. The data are not publicly available due to the privacy of the subjects.

## Supplementary Materials

The following supporting information can be downloaded at: https://www.mdpi.com/article/10.3390/e26090769/s1.

## References

[1] Paulus F.W., Ohmann S., von Gontard A., Popow C. (2018). Internet gaming disorder in children and adolescents: A systematic review. Dev. Med. Child. Neurol..

[2] American Psychiatric Association (2013). Diagnostic and Statistical Manual of Mental Disorders.

[3] Aarseth E., Bean A.M., Boonen H., Carras M.C., Coulson M., Das D., Deleuze J., Dunkels E., Edman J., Ferguson C.J. (2017). Scholars’ open debate paper on the World Health Organization ICD-11 Gaming Disorder proposal. J. Behav. Addict..

[4] NHS, Central and North West London. https://www.cnwl.nhs.uk/national-centre-gaming-disorders.

[5] Kagawa Prefectural Government Kagawa Prefecture Ordinance on Internet and Game Addiction. https://www.pref.kagawa.lg.jp/documents/10293/0324gj24.pdf.

[6] Yen J.-Y., Lin P.-C., Wu H.-C., Ko C.-H. (2022). The withdrawal-related affective, gaming urge, and anhedonia symptoms of internet gaming disorder during abstinence. J. Behav. Addict..

[7] Park S.M., Lee J.Y., Choi A.R., Kim B.M., Chung S.J., Park M., Kim I.Y., Park J., Choi J., Hong S.J. (2020). Maladaptive neurovisceral interactions in patients with internet gaming disorder: A study of heart rate variability and functional neural connectivity using the graph theory approach. Addict. Biol..

[8] Kim N., Hughes T.L., Park C.G., Quinn L., Kong I.D. (2016). Altered autonomic functions and distressed personality traits in male adolescents with internet gaming addiction. Cyberpsychol. Behav. Soc. Netw..

[9] Hong S.J., Lee D., Park J., Namkoong K., Lee J., Jang D.P., Lee J.E., Jung Y.-C., Kim I.Y. (2018). Altered heart rate variability during gameplay in internet gaming disorder: The impact of situations during the game. Front. Psychiatry.

[10] Lee D., Hong S.J., Jung Y.-C., Park J., Kim I.Y., Namkoong K. (2016). Altered heart rate variability during gaming in internet gaming disorder. Cyberpsychol. Behav. Soc. Netw..

[11] Long K., Zhang X., Wang N., Lei H. (2024). Heart rate variability during online video game playing in habitual gamers: Effects of internet addiction scale, ranking score and gaming performance. Brain Sci..

[12] Lee D., Park J., Namkoong K., Hong S.J., Kim I.Y., Jung Y.-C. (2021). Diminished cognitive control in Internet gaming disorder: A multimodal approach with magnetic resonance imaging and real-time heart rate variability. Prog. Neuropsychopharmacol. Biol. Psychiatry.

[13] Schulz S., Haueisen J., Bär K.-J., Voss A. (2020). The cardiorespiratory network in healthy first-degree relatives of schizophrenic patients. Front. Neurosci..

[14] Schulz S., Bär K.-J., Voss A. (2015). Analyses of heart rate, respiration and cardiorespiratory coupling in patients with schizophrenia. Entropy.

[15] Zhao L., Yang L., Su Z., Liu C. (2019). Cardiorespiratory coupling analysis based on entropy and cross-entropy in distinguishing different depression stages. Front. Physiol..

[16] Zanetti M., Faes L., Nollo G., Cecco M.D., Pernice R., Maule L., Pertile M., Fornaser A. (2019). Information dynamics of the brain, cardiovascular and respiratory network during different levels of mental stress. Entropy.

[17] Udhayakumar R., Rahman S., Buxi D., Macefield V.G., Dawood T., Mellor N., Karmakar C. (2023). Measurement of stress-induced sympathetic nervous activity using multi-wavelength PPG. R. Soc. Open Sci..

[18] Stautland A., Jakobsen P., Fasmer O.B., Osnes B., Torresen J., Nordgreen T., Oedegaard K.J. (2023). Reduced heart rate variability during mania in a repeated naturalistic observational study. Front. Psychiatry.

[19] Toschi-Dias E., Tobaldini E., Solbiati M., Costantino G., Sanlorenzo R., Doria S., Irtelli F., Mencacci C., Montano N. (2017). Sudarshan Kriya yoga improves cardiac autonomic control in patients with anxiety-depression disorders. J. Affect. Disord..

[20] Lehrer P., Buckman J.F., Mun E.-Y., Vaschillo E.G., Vaschillo B., Udo T., Ray S., Nguyen T., Bates M.E. (2013). Negative mood and alcohol problems are related to respiratory dynamics in young adults. Appl. Psychophysiol. Biofeedback.

[21] Huang P.-H., Hsiao T.-C. (2023). Intrinsic entropy: A novel adaptive method for measuring the instantaneous complexity of time series. IEEE Signal Process Lett..

[22] Huang P.-H., Hsiao T.-C. (2024). Use of intrinsic entropy to assess the instantaneous complexity of thoracoabdominal movement patterns to indicate the effect of the iso-volume maneuver trial on the performance of the step test. Entropy.

[23] Elstad M., O’Callaghan E.L., Smith A.J., Ben-Tal A., Ramchandra R. (2018). Cardiorespiratory interactions in humans and animals: Rhythms for life. Heart Circ. Physiol..

[24] Statista Distribution of Video Game Users in the United States as of June 2024, by Age Group. https://www.statista.com/forecasts/1277856/video-game-users-age-market-usa.

[25] Statista Share of Mobile Gamers in Southeast Asia in 2021, by Country and Age. https://www.statista.com/forecasts/1303533/southeast-asia-number-of-mobile-gamers-by-country-and-age.

[26] Chen S.-H., Weng L.-J., Su Y.-J., Wu H.-M., Yang P.-F. (2003). Development of Chinese Internet addiction scale and its psychometric study. Chin. J. Psychol..

[27] Petry N.M., Rehbein F., Gentile D.A., Lemmens J.S., Rumpf H.-J., Mößle T., Bischof G., Tao R., Fung D.S.S., Borges G. (2014). An international consensus for assessing internet gaming disorder using the new DSM-5 approach. Addiction.

[28] Ko C.-H., Yen C.-F., Yen C.-N., Yen J.-Y., Chen C.-C., Chen S.-H. (2005). Screening for internet addiction: An empirical study on cut-off points for the Chen internet addiction scale. Kaohsiung J. Med. Sci..

[29] Bradley M.M., Lang P.J. (1994). Measuring emotion: The self-assessment manikin and the semantic differential. J. Behav. Ther. Exp. Psychiatry.

[30] Chen Y.-C., Hsiao T.-C., Hsu J.-H., Chen J.-L. (2014). Breathing pattern recognition of abdominal wall movement by using ensemble empirical mode decomposition. Adv. Adapt. Data Anal..

[31] Ji H.-M., Hsiao T.-C. (2019). A novel cue-induced abdominal reaction analysis for internet gaming disorder. J. Med. Syst..

[32] Ji H.-M., Chen L.-Y., Hsiao T.-C. Real-time detection of internet addiction using reinforcement learning system. Proceedings of the Genetic and Evolutionary Computation Conference.

[33] Chi H.-M., Hsiao T.-C. (2021). Extended classifier system with continuous real-coded variables for feature extraction of instantaneous pulse-rate variability and respiration of individuals with gaming disorder. BioMed Eng. Online.

[34] Yeh J.-R., Shieh J.-S., Huang N.E. (2010). Complementary ensemble empirical mode decomposition: A novel noise enhanced data analysis method. Adv. Adapt. Data Anal..

[35] Richman J.S., Moorman J.R. (2000). Physiological time-series analysis using approximate entropy and sample entropy. Heart Circ. Physiol..

[36] Platiša M.M., Radovanović N.N., Kalauzi A., Milašinović G., Pavlović S.U. (2020). Multiscale entropy analysis: Application to cardio-respiratory coupling. Entropy.

[37] Valente M., Javorka M., Porta A., Bari V., Krohova J., Czippelova B., Turianikova Z., Nollo G., Faes L. (2018). Univariate and multivariate conditional entropy measures for the characterization of short-term cardiovascular complexity under physiological stress. Physiol. Meas..

[38] Hagemann D., Waldstein S.R., Thayer J.F. (2003). Central and autonomic nervous system integration in emotion. Brain Cogn..

[39] Palaus M., Marron E.M., Viejo-Sobera R., Redolar-Ripoll D. (2017). Neural basis of video gaming: A systematic review. Front. Hum. Neurosci..

[40] King D.L., Herd M.C.E., Delfabbro P.H. (2017). Tolerance in internet gaming disorder: A need for increasing gaming time or something else?. J. Behav. Addict..

[41] Kim H., Ha J., Chang W.-D., Park W., Kim L., Im C.-H. (2018). Detection of craving for gaming in adolescents with internet gaming disorder using multimodal biosignals. Sensors.

[42] Solá-Soler J., Cuadros A., Giraldo B.F. Cardiorespiratory phase synchronization increases during certain mental stimuli in healthy subjects. Proceedings of the 40th Annual International Conference of the IEEE Engineering in Medicine and Biology Society.

[43] Chang J.S., Kim E.Y., Jung D., Jeong S.H., Kim Y., Roh M.-S., Ahn Y.M., Hahm B.-J. (2015). Altered cardiorespiratory coupling in young male adults with excessive online gaming. Biol. Psychol..

[44] Coyne S.M., Dyer W.J., Densley R., Money N.M., Day R., Harper J.M. (2015). Physiological indicators of pathologic video game use in adolescence. J. Adolesc. Health.

[45] Yen J.-Y., Yeh Y.-C., Wang P.-W., Liu T.-L., Chen Y.-Y., Ko C.-H. (2018). Emotional regulation in young adults with internet gaming disorder. Int. J. Environ. Res. Public Health.

[46] Schulz S., Haueisen J., Bär K.-J., Voss A. (2019). Altered causal coupling pathways within the central-autonomic-network in patients suffering from schizophrenia. Entropy.

[47] Chen Y.-F., Huang X.-Y., Chien C.-H., Cheng J.-F. (2017). The Effectiveness of diaphragmatic breathing relaxation training for reducing anxiety. Perspect. Psychiatr. Care.

[48] Noble D.J., Hochman S. (2019). Hypothesis: Pulmonary afferent activity patterns during slow, deep breathing contribute to the neural induction of physiological relaxation. Front. Physiol..

[49] Zaccaro A., Piarulli A., Laurino M., Garbella E., Menicucci D., Neri B., Gemignani A. (2018). How breath-control can change your life: A systematic review on psycho-physiological correlates of slow breathing. Front. Hum. Neurosci..

[50] Dick T.E., Mims J.R., Hsieh Y.-H., Morris K.F., Wehrwein E.A. (2014). Increased cardio-respiratory coupling evoked by slow deep breathing can persist in normal humans. Respir. Physiol. Neuro.

[51] Martínez-Hernández V.J., Dorantes-Méndez G. Analysis of cardiovascular and cerebral interactions in response to cognitive stressors stimulus. Proceedings of the XLVI Mexican Conference on Biomedical Engineering.

[52] Chi H.-M., Hsiao T.-C. (2023). Effect of short-term abdominal breathing on heart rate variability as an indicator of emotional regulation in college student with internet gaming disorder. Cogent Psychol..

[53] Young K.S., Brand M. (2017). Merging theoretical models and therapy approaches in the context of internet gaming disorder: A personal perspective. Front. Psychol..

[54] Yao Y.-W., Chen P.-R., Li C.-S.R., Hare T.A., Li S., Zhang J.-T., Liu L., Ma S.-S., Fang X.-Y. (2017). Combined reality therapy and mindfulness meditation decrease intertemporal decisional impulsivity in young adults with Internet gaming disorder. Comput. Hum. Behav..

[55] Govindan R.B., Wilson J.D., Eswaran H., Lowery C.L., Preißl H. (2007). Revisiting sample entropy analysis. Phys. A Stat. Mech. Appl..

