# Supplementary material for: Physiological Regularity and Synchrony in Individuals with Gaming Disorder
Source: Entropy (Basel). 2024 Sep 8;26(9):769. doi: 10.3390/e26090769 (PMC11431265; doi:10.3390/e26090769)
Supplement: Supplementary file 1 [file entropy-26-00769-s001.zip › entropy-3086040-supplementary.pdf]

Table S1. the mean ± standard deviation of physiological complexity and synchrony.

Parameter	Group	Before BE				After BE			
		baseline	game1	game2	recovery	baseline	game1	game2	recovery
SE_{PW}	HC	0.36 ± 0.07	0.42 ± 0.10	0.41 ± 0.08	0.38 ± 0.08	0.39 ± 0.06	0.42 ± 0.06	0.40 ± 0.06	0.39 ± 0.05
	GD	0.37 ± 0.07	0.41 ± 0.07	0.40 ± 0.07	0.38 ± 0.07	0.39 ± 0.06	0.41 ± 0.07	0.41 ± 0.07	0.40 ± 0.06
SE_{TWM}	HC	0.11 ± 0.04	0.20 ± 0.06	0.19 ± 0.06	0.10 ± 0.04	0.12 ± 0.05	0.17 ± 0.05	0.18 ± 0.04	0.12 ± 0.05
	GD	0.13 ± 0.06	0.19 ± 0.09	0.20 ± 0.08	0.10 ± 0.04	0.12 ± 0.04	0.16 ± 0.04	0.18 ± 0.06	0.11 ± 0.04
SE_{AWM}	HC	0.12 ± 0.04	0.22 ± 0.05	0.22 ± 0.05	0.12 ± 0.04	0.13 ± 0.05	0.20 ± 0.04	0.21 ± 0.05	0.13 ± 0.04
	GD	0.12 ± 0.03	0.20 ± 0.07	0.22 ± 0.07	0.11 ± 0.04	0.13 ± 0.04	0.19 ± 0.07	0.20 ± 0.07	0.13 ± 0.04
CSE_{PW-TWM}	HC	0.93 ± 0.13	0.82 ± 0.26	0.84 ± 0.19	0.99 ± 0.23	0.97 ± 0.11	0.91 ± 0.19	0.88 ±0.14	0.97 ± 0.16
	GD	1.05 ± 0.30	0.98 ± 0.21	1.04 ± 0.48	1.03 ± 0.16	1.00 ± 0.22	1.00 ± 0.24	0.95 ± 0.20	1.07 ± 0.22
CSE_{PW-AWM}	HC	0.89 ± 0.12	0.78 ± 0.18	0.80 ± 0.16	0.87 ± 0.10	0.91 ± 0.12	0.82 ± 0.12	0.82 ± 0.15	0.88 ± 0.11
	GD	1.02 ± 0.26	0.95 ± 0.27	0.88 ± 0.14	0.97 ± 0.11	0.97 ± 0.23	0.93 ± 0.25	0.90 ± 0.25	0.96 ± 0.21
$CSE_{TWM-AWM}$	HC	0.12 ± 0.04	0.22 ± 0.04	0.22 ± 0.04	0.12 ± 0.04	0.13 ± 0.04	0.19 ± 0.04	0.20 ± 0.04	0.13 ± 0.04
	GD	0.14 ± 0.09	0.21 ± 0.07	0.22 ± 0.06	0.11 ± 0.03	0.13 ± 0.03	0.19 ± 0.05	0.19 ± 0.07	0.12 ± 0.04

BE, breathing exercise; CSE_{PW-TWM}, Cross-Sample Entropy (CSE) of between pulse wave (PW) and thoracic wall movement (TWM) signals; CSE_{PW-AWM}, CSE of between PW and abdominal wall movement (AWM) signals; $CSE_{TWM-AWM}$, CSE of between TWM and AWM signals; SE_{AWM}, Sample Entropy (SE) of AWM signal; SE_{PW}, SE of PW signal; SE_{TWM}, SE of TWM signal
